# Treating Cocaine Addiction, Obesity, and Emotional Disorders by Viral Gene Transfer of Butyrylcholinesterase

**DOI:** 10.3389/fphar.2018.00112

**Published:** 2018-02-27

**Authors:** Stephen Brimijoin, Yang Gao, Liyi Geng, Vicky P. Chen

**Affiliations:** Department of Molecular Pharmacology and Experimental Therapeutics, Mayo Clinic, Rochester, MN, United States

**Keywords:** butyrylcholinesterase, cocaine, ghrelin, addiction, obesity, stress, viral gene transfer

## Abstract

Butyrylcholinesterase (BChE), a plasma enzyme that hydrolyses the neurotransmitter, acetylcholine relatively well, with far lower efficiency than acetylcholinesterase (AChE) but with the capability to degrade a broad range of bioactive esters. AChE is universally understood as essential to cholinergic neurotransmission, voluntary muscle performance, and cognition, among other roles, and its catalytic impact is essential for life. A total absence of BChE activity, whether by enzyme inhibition or simple lack of enzyme protein is not only compatible with life, but does not lead to obvious physiologic disturbance. However, very recent studies at Mayo Clinic have amassed support for the concept that BChE does have a true physiological role as a “ghrelin hydrolase” and, pharmacologically, as a cocaine hydrolase. Human subjects and animal mutations that lack functional BChE show higher than normal levels of ghrelin, an acylated peptide that drives hunger and feeding, along with certain emotional behaviors. Mice treated by viral gene transfer of BChE show higher plasma levels of enzyme and lower levels of ghrelin. Ghrelin is acknowledged as a driver of food-seeking and stress. This brief review examines some key phenomena and considers means of modulating BChE as treatments for cocaine addiction, anxiety, aggression, and obesity.

## Introduction

This brief review focuses on unexpected discoveries in our laboratory during long-term manipulation of plasma butyrylcholinesterase (BChE) by viral gene transfer for multiple purposes. That blood-borne enzyme, nearly universal in higher vertebrates, was first identified as a cholinesterase 70 years ago by Augustinsson who established its ability to hydrolyze acetylcholine (Augustinsson, 1948). However, this activity was deemed physiologically irrelevant because it was soon recognized that individuals who entirely lack BChE show no discernable disability (Manoharan et al., 2007), thus presenting a dramatic contrast with acetylcholinesterase, which is essential to life. In the course of time it became clear that BChE was efficient in hydrolyzing certain other bioactive esters, including butyrylcholine and succinylcholine. Neither of these substrates are found in nature, but the latter was once widely used to achieve transient muscle relaxation that is helpful in abdominal surgery. Succinylcholine’s surgical utility depended on rapid hydrolysis by BChE (Evans et al., 1952). Circulating levels of the plasma enzyme in most patients would hydrolyze the muscle relaxant so readily that abdominal tension could be modulated by the rate of drug infusion. Equally important, full respiratory control was typically restored before recovery from anesthesia. Unfortunately, though, some patients carry a genetic variant of BChE that is far less efficient with succinylcholine (Viby-Mogensen and Hanel, 1978). Before this fact was recognized, several cases led to severe anoxia and brain damage from prolonged apnea. That extremely unwelcome outcome was a principal driver leading to the new fields of pharmacokinetics and pharmacogenomics. However, despite active investigations over several decades, no plausible role for BChE in *normal* physiology had emerged until very recently, for lack of a bioactive ester other than acetylcholine to serve as a plausible target. This state of knowledge continued until Devries reported that BChE hydrolyzes ghrelin, the so-called “hunger hormone” (De Vriese et al., 2004), cleaving its unique octanoyl group esterified on the serine-3 residue of the active peptide. That result was confirmed by Schopfer et al. (2015) but the reaction was very slow, orders of magnitude lower than with typical small-molecule esters. It was difficult to believe it could be physiological relevant. Understandably, most investigators rejected the idea.

**FIGURE 1:**
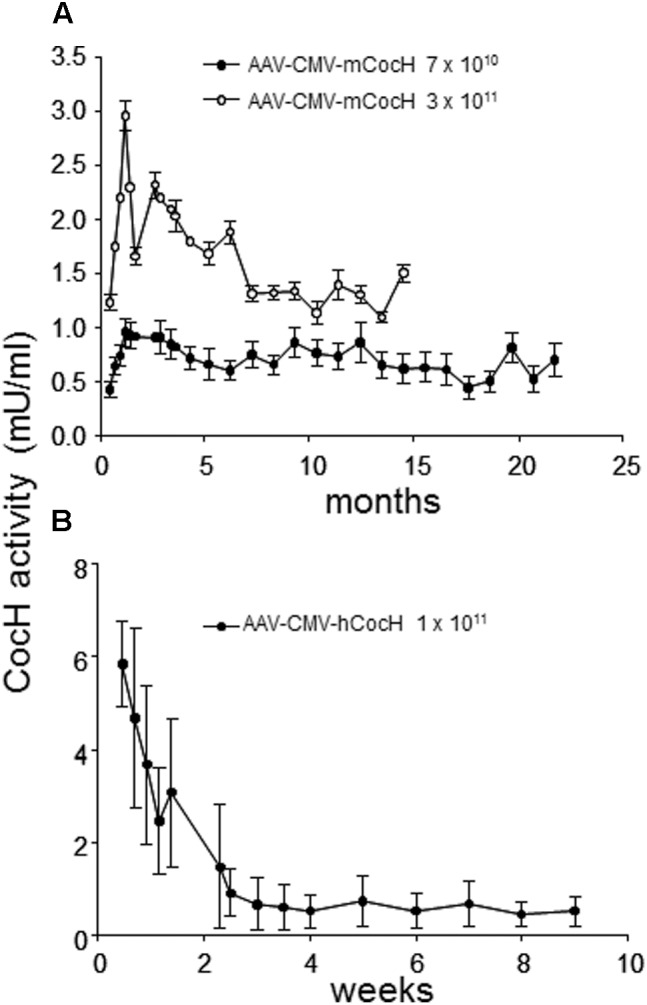
Time course of plasma cocaine hydrolysis activities in mice receiving viral gene transfer of mouse or human BChE mutated for enhanced cocaine hydrolysis (CocH by i.v. injection of standard AAV). Data are from repeated samples of mouse plasma across time (weeks to months as noted). CocH activity was measured by enzymatic release of tritium-label isolated by solvent separation (Brimijoin et al., 2002). **(A)** Mice given AAV gene transfer of mouse CocH. Levels of expression were modest but were maintained for more than 1 year. **(B)** Mice given gene transfer of human CocH. Activities were higher, but much shorter lived (Geng et al., 2013, Figure 2).

**FIGURE 2:**
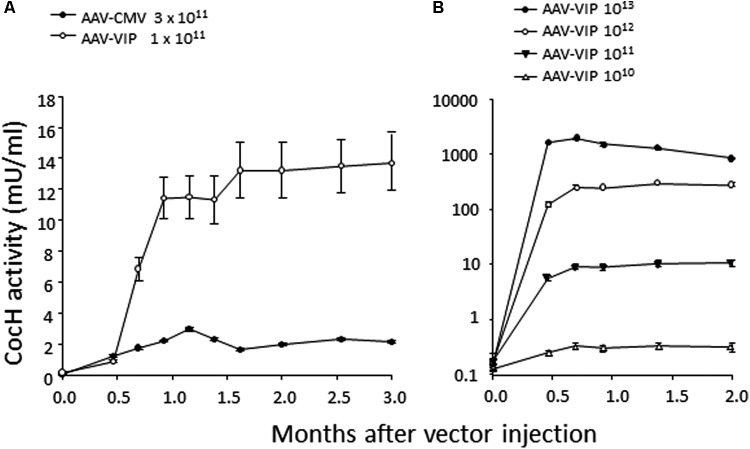
Time course and dose-response measures of *in vivo* cocaine hydrolysis activity in mice given gene transfer of mutant mouse CocH with different viral vectors. **(A)** “Standard AAV vector” and **(B)** high doses of “AAV-VIP” vector. Note the log-linear 10-fold increments of hydrolysis matching the log-linear increments of vector (Geng et al., 2013, Figure 4).

This state of affairs persisted until our own research encountered unexpected results while pursuing rodent studies on the toxicity and safety of BChE gene transfer as a potential therapy for cocaine abuse. It was well-known that native BChE hydrolyzes cocaine (Gorelick, 1997). Actually, it is a major factor in cocaine metabolism by humans and rodents. Normal BChE levels in liver and plasma are enough to limit cocaine-induced euphoria to just a few minutes after drug intake. This feature encouraged exploration of treatments for cocaine addiction and overdose, by raising BChE blood levels and enhancing its efficiency by modifying key residues in and around the enzyme’s catalytic site. Accordingly, we and others utilized computer-based simulations of “drug-protein docking” to guide mutations likely to remove obstacles for cocaine access and proper orientation in the catalytic gorge. Those efforts turned out to be surprisingly effective. At Mayo with Pang and colleagues we used computational biology, enzyme expression, and enzyme assay to achieve a 20-fold enhancement in BChE’s rate of cocaine hydrolysis (Sun et al., 2002). Soon afterward, Zhan and colleagues at the University of Kentucky discovered several additional mutations that could be combined to raise catalytic efficiency for cocaine by approximately 1000-fold (Yang et al., 2010). The fully optimized enzyme deserves to be considered as a true cocaine hydrolase, “CocH.” Not surprisingly, delivering enzyme protein to mice by direct injection proved highly effective in reducing cocaine’s behavioral impact, as measured in reduced drug-stimulated motor activity (Brimijoin and Gao, 2012; Murthy et al., 2014; Zlebnik et al., 2014). Mice treated with CocH protein at a level of 5 mg/kg were able to tolerate cocaine doses that would normally induce massive convulsions followed by death within minutes after drug injection (Xue et al., 2013). In fact, those animals merely sat calmly grooming themselves, as if nothing happened. We then moved to viral gene transter on an AAV-CMV vector platform. It was to be expected that species immunogenicity would affect the expression period. Indeed, mice transduced with human CocH showed curtailed expression compared to those given *mouse* CocH (**Figure 1**). We enhanced vector expression efficiency by optimizing promotor elements. An AAV-VIP vector was more effective than AAV-CMV (**Figure 2A**). It raised plasma levels of CocH activity in a dose-dependent fashion and sustained a 10-fold increase for an entire 3-month period of observation (**Figure 2B**). These outcomes encouraged further study of CocH gene transfer to determine if it could deliver equivalent amounts of enzyme for a year or more. If so, it might enable “newly clean cocaine addicts” to escape typical relapse into drug-taking.

Before attempting a move toward human studies with this concept, it was essential to explore potential risks inherent in vector-based delivery of mutated BChE. Fortunately, support from the National Institute of Drug Abuse (NIDA) enabled funding for animal toxicology and safety studies of this concept. Initial short-term observations had revealed no adverse effects, but long term data were lacking. Therefore studies were launched with group-housed male Balb/c and C57 black mice, untreated and vector-treated, and ran for over a year, essentially a mouse’s lifetime. At first, no apparent effects of any sort emerged. But as time elapsed, our animal care personnel began to notice mice fighting in their group cages and required them to be put into single animal cages (with a large rise in housing cost). After numerous such alerts, one of us (GY), realized that this problem was limited to the *control animals*. They were prone to fighting. They were battered and bitten, with ragged coats of prematurely gray fur (Chen et al., 2015). In stark contrast, few if any vector-treated mice showed any such damage. While we watched those healthy group-housed animals closely, they appeared to be “just playing with each other.” In other words, it seemed that the vector treatment had modulated aggressive impulses in these mice and/or reduced stress and anxiety. Ultimately, the BChE-enhanced mice outlived the controls. Clearly, vector treatment in this model was not toxic. Faced with that outcome we hypothesized that high BChE levels in the active-vector mice were modulating a stress hormone. Since ghrelin is linked to hunger and anxiety, and its serine-3 ester is key to function, it seemed likely to be a driver of the behavioral differences between mice with high or absent BChE despite the enzyme’s poor catalytic activity with that hormone.

We soon replicated the earlier *in vitro* findings that BChE hydrolyzes ghrelin. Its hydrolysis of this substrate was several orders of magnitude slower than with acetylcholine, but treating mice with an organophosphate anticholinesterase to inhibit all BChE activity led to a large rise in plasma levels of the peptide hormone. Conversely, raising mouse BChE levels by gene transfer with an AAV8 viral expression vector led to sustained decreases in plasma ghrelin, on the order of 80% or greater. These results led us to conclude that BChE is indeed a natural regulator of ghrelin and almost certainly the major one. In that role the enzyme has a broad impact on behaviors and emotional states driven or affected by that hormone. It now seems clear that BChE is truly a natural “ghrelin hydrolase,” and the BChE-ghrelin system is an axis that impacts many ordinary but vital states and processes including hunger, food-seeking, stress, anxiety, fear, and aggression (Brimijoin et al., 2016).

Our aggression findings appear broadly in line with recent literature showing that ghrelin plays important roles in regulating mood-related behaviors such as stress and anxiety (Chuang and Zigman, 2010; Bali and Jaggi, 2016). However, the mechanism by which ghrelin affects these behaviors is not yet fully elucidated. Some studies report that ghrelin relieves anxiety and defends against stress-induced depressive symptomes (Lutter et al., 2008; Spencer et al., 2012), while others have reported that ghrelin enhances fear and anxiety after acute and chronic stress exposure (Carlini et al., 2002; Meyer et al., 2014). The latter sudies support our finding that chronic reduction of plasma ghrelin promotes lower aggression. Plausible explanations for these divergent outcomes could include the following: (1) Various animal models of emotional states bear inherent differences in underlying neural pathways; (2) Ghrelin receptor is an unusual receptor that is self- activated, with high constitutive signaling in the absence of ligand, and self-desensitizing.

Because ghrelin is thought to enhance the reward value of certain drugs, reduced levels of that hormone may also reduce the strength of a cocaine-induced reward. In fact, numerous studies have shown that central ghrelin signaling also modulates cocaine-induced reward. Elevated plasma levels of ghrelin are associated with cocaine-seeking behavior (Tessari et al., 2007). Peripheral ghrelin administration augments cocaine-induced locomotor action and conditioned place preference (Wellman et al., 2005; Davis et al., 2007). Pharmacological or genetic suppression of ghrelin or ghrelin recepotor attenuates the cocaine-induced reward (Abizaid et al., 2011; Clifford et al., 2011; Jang et al., 2013).

At this point it should be emphasized that BChE-driven ghrelin hydrolysis is quite slow. Thus, the enzyme is unlikely to affect the impact of phasic ghrelin release into the circulation or extracellular fluid in tissue compartments, either from the stomach or from the brain. Instead, it is likely that BChE’s most important physiological role is to lower background levels of ghrelin in the brain, where its target, the growth hormone secretagogue receptor, exists in a chronic state of partial desensitization (Mear et al., 2013). In relieving that state, BChE will enhance ghrelin’s phasic receptor impact.

**FIGURE 3:**
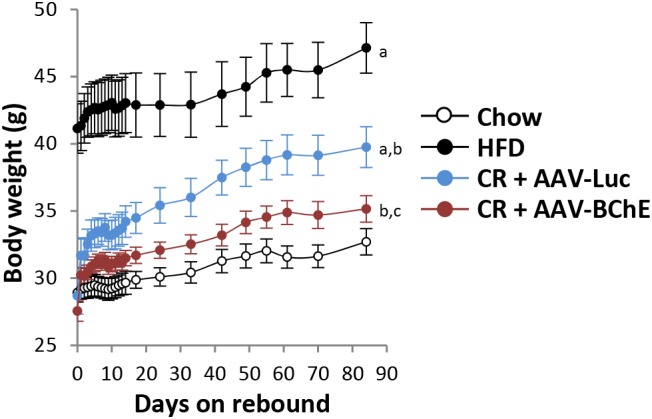
Rebound weight gain in formerly obese mice on high fat diet after return to free access standard mouse chow. (a) Significant with respect to chow group; (b) significant with respect to HFD group; (c) significant with respect to CR + AAV-Luc group. Significance was set at *P* < 0.05 by two-way repeated-measures ANOVA Chen et al. (2017).

In humans, plasma BChE levels drop during protein-energy malnutrition (Lampon et al., 2012) but they are consistently elevated in populations that are obese or diabetic (Abbott et al., 1993). In addition, plasma BChE activity correlates positively with levels of serum triglyceride, total cholesterol, fasting insulin, and insulin resistance, but it correlates negatively with high density lipoprotein-cholesterol (Iwasaki et al., 2007; Santarpia et al., 2013). Bearing these facts in mind, it seemed worthwhile to determine if sustained elevation of BChE could be effective in treating obesity. The key question was, would it blunt the intense food cravings that drive seemingly inevitable rebound weight gain after a long supervised diet has returned an obese person to healthy body weight? Addressing this issue in a recent publication (Chen et al., 2017), we assigned C57BL mice to two treatment groups for gene transfer with AAV8 viral vector. The vector for Group 1 encoded BChE while that for Group 2, controls, encoded the irrelevant protein, firefly luciferase. All mice were initially given unrestricted access to attractive high-fat diet and both groups became obese within 3 months. At that point, the animals were switched to low-fat (standard) mouse chow in limited quantity until they returned to their initial body weight at study entry. In the final phase, both groups were allowed unlimited access to the low-fat food and followed for a further 3 months. On that regime, all mice regained some weight, but none of the BChE-treated mice returned to obesity whereas all the control mice did so (**Figure 3**). It remains to be seen if similar outcomes can be achieved in treatment-seeking humans, but the prospects for success seem very promising in our view, if the AAV vector treatment and elevated BChE levels prove as safe as we expect.

One last concern for risks in treatments based on prolonged elevation of BChE levels, is the nearly ubiquitous findings of BChE deposits in the senile plaques of brains in patients who die with Alzheimer’s disease (Guillozet et al., 1997; Darvesh et al., 2010). Strikingly similar patterns are also found in brains of transgenic mice engineered to express human genes for beta amyloid and other factors that promote senile plaques, neuronal loss, and cognitive impairment (Elder et al., 2010). From these observations, prominent Alzheimer experts have concluded that BChE-amyloid deposits represent an important factor driving disease progression. However, current data leave room for a completely opposite interpretation: BChE is an amyloid-binding factor that at least partially *protects* brain neurons by *reducing* amyloid aggregation and toxicity. *In vitro* studies by Hermona Soreq at the University of Jerusalem support that concept, showing that BChE’s presence in a solution of amyloid-beta monomers greatly retards and reduces beta-amyloid aggregation (Diamant et al., 2006). We are presently conducting experiments to determine whether increased brain BChE in AD mice, driven by viral gene transfer of the enzyme, will accelerate or depress amyloid plaque formation and maze learning. This is a long-term project but, in the animals tested so far, there has been no increase in plaque burden. Neither have we seen any enzyme-induced enhanced loss of cognitive function over time as assessed in multiple tests with the standard “Novel Object Paradigm.”

## Conclusion

We end by stressing that BChE is a near ubiquitous enzyme in mammals, and, as is commonly the case, evolutionary processes over time have found multiple uses for this protein. One of these is certainly to protect plant-eating animals from bioactive esters in foods that might otherwise be toxic. A second key role is to serve as a ghrelin regulator. Because of ghrelin’s widespread impact on important emotional and physiological phenomena, and BChE’s apparent role in regulating ghrelin’s activity, extensive further research is warranted to pursue the enzyme’s influences on food intake, obesity, anxiety, and stress. In addition, we recognize that strong efforts should be made to assess the potential for long term ghrelin reduction to impact ghrelin’s neuroprotective role in brain.

## Author Contributions

SB prepared the first rough draft and polished the final draft. YG and LG contributed numerous details and VC completed the full details and helped with multiple revisions.

## Conflict of Interest Statement

The authors declare that the research was conducted in the absence of any commercial or financial relationships that could be construed as a potential conflict of interest.
